# Novel Inhibitor-Based Therapies for Thyroid Cancer—An Update

**DOI:** 10.3390/ijms222111829

**Published:** 2021-10-31

**Authors:** Maciej Ratajczak, Damian Gaweł, Marlena Godlewska

**Affiliations:** 1 Centre of Postgraduate Medical Education, Department of Endocrinology, Marymoncka 99/103, 01-813 Warsaw, Poland; maciej.ratajczak@cmkp.edu.pl; 2 Centre of Postgraduate Medical Education, Department of Immunohematology, Marymoncka 99/103, 01-813 Warsaw, Poland; 3 Centre of Postgraduate Medical Education, Department of Biochemistry and Molecular Biology, Marymoncka 99/103, 01-813 Warsaw, Poland

**Keywords:** thyroid cancer, targeted therapies, kinase inhibitors, RAI-refractory

## Abstract

Thyroid cancers (TCs) are the most common tumors of the endocrine system and a constant rise in the number of TC cases has been observed for the past few decades. TCs are one of the most frequent tumors in younger adults, especially in women, therefore early diagnosis and effective therapy are especially important. Ultrasonography examination followed by fine needle biopsy have become the gold standard for diagnosis of TCs, as these strategies allow for early-stage detection and aid accurate qualification for further procedures, including surgical treatment. Despite all the advancements in detection and treatment of TCs, constant mortality levels are still observed. Therefore, a novel generation line of targeted treatment strategies is being developed, including personalized therapies with kinase inhibitors. Recent molecular studies on TCs demonstrate that kinase inhibitor-based therapies might be considered as the most promising. In the past decade, new kinase inhibitors with different mechanisms of action have been reported and approved for clinical trials. This review presents an up-to-date picture of new approaches and challenges of inhibitor-based therapies in treatment of TCs, focusing on the latest findings reported over the past two years.

## 1. Introduction

### 1.1. Thyroid Cancer: Origins and Classification

The thyroid gland consists of two types of cells: thyrocytes (epithelial, follicular cells) and C cells (parafollicular cells). Thyrocytes form thyroid follicles, which are the structural and functional units of the mature thyroid gland. Thyroid hormones (THs), which are involved in regulation of metabolism, are synthetized and stored in the follicular lumen. Production and secretion of THs is controlled by the thyroid-stimulating hormone (TSH) [1]. C cells are located adjacently to thyroid follicles and are responsible for production of various biologically-active substances, such as calcitonin, carcinoembryonic antigen, prostaglandins and serotonin [2].

Classification of thyroid cancer is based on the histological type of thyroid cells. Differentiated thyroid cancer (DTC) and de-differentiated thyroid cancer (DeTC) arise from follicular cells. DTC is subclassified into papillary thyroid carcinoma (PTC), follicular thyroid carcinoma (FTC) and Hürtle cell carcinoma (HCC) [3]. HCC, which was previously recognized as FTC, is now classified as a subtype of DTC [3,4]. The rarest, but most aggressive forms of TC, anaplastic thyroid carcinoma (ATC) and poorly differentiated thyroid carcinoma (PDTC) are classified as DeTCs. PDTCs are a subset of thyroid tumors intermediate between DTCs and ATCs. Medullary thyroid cancer (MTC) is classified as the forth main type of TC and in contrast to DTC and DeTC, is a neuroendocrine tumor originating from parafollicular C cells. The Cancer Genome Atlas (TCGA) Research Network [5] data and other subsequent studies [6,7,8] underlined the need for reclassification of thyroid cancers into molecular- and genotype-based subtypes that would predict their prognosis and be helpful in administration of treatment strategies. Therefore, molecular and genetic aberrations were additionally considered and implemented to the World Health Organization (WHO) Classification of Tumours of Endocrine Organs that was published in 2017 [3].

### 1.2. Thyroid Cancer Epidemiology

Thyroid cancers (TCs) account for ~3% of all malignancies worldwide. Among cancers of the endocrine system, TCs are the most common (approximately 95% of all cases). For the past several decades, a substantial increase in cancer morbidity has been observed and the same tendency has been found for TCs. According to GLOBOCAN 2020, the WHO cancer statistics for 185 countries worldwide, 586,202 new TC cases were reported with mortality of all cancer types reaching 0.4% [9]. This ranks TCs in the 11th place for all cancer incidences. Until 2020, the morbidity of TCs was the most rapidly increasing among all cancers in the United States. In 2021, in the USA alone, the number of TCs was estimated to be about 44,280 cases and ~2200 patients were expected to die [10].

DTCs account for more than 90% of all TC cases, where ~84% of incidences are PTCs, while diagnoses of FTCs and HCCs stand for approximately 2% each. ATC also represents ~2% of thyroid malignancies, and as the most aggressive thyroid tumor, is responsible for 50% of annual thyroid cancer deaths. ATC generally affects 60–70 year-old patients. PDTC is a relatively rare disease, accounting for slightly more than 2% of all new cases of TCs each year. MTC accounts for about 4–5% of thyroid cancers. Most MTCs (70–80%) are observed in 50 to 60 year-old patients and result from sporadic mutations. Over 50 different point mutations have been linked with development of MTC [11]. A hereditary pattern of MTC is present in 20–30% of cases, transmitted as an autosomal dominant trait, while a mutation in the *RET* gene at codon 634 is responsible for development of familial MTC in 85% of patients. MTC is associated with multiple endocrine neoplasia (MEN) type IIA and IIB syndrome. Approximately 35% of patients with MTC have lymph node metastases at the time of diagnosis, while about 20% have distant metastases [3,12,13].

### 1.3. Thyroid Cancer: Risk Factors

Incidences of TC are most common among younger adults [14,15]. Women are three times more likely to develop TC than men [10,16]. This sex-dependent disparity is mostly observed for PTCs and FTCs [17]. The possible relationship between female hormonal and reproductive processes (menstrual cycle, pregnancy, menopause and hormone replacement therapy) has been studied, but no direct correlation with higher risk of developing thyroid cancer was established [18,19]. According to the American Thyroid Association (ATA), a high-risk factors for TC are: (i) history of TC in one or more first degree relatives, (ii) exposure to external beam radiation in the childhood, (iii) exposure to ionizing radiation in the childhood or adolescence, (iv) prior hemithyroidectomy with discovery of TC, (v) ^18^F-fluoro-2-deoxy-D-glucose (^18^FDG) avidity on positron emission tomography (PET) scanning in thyroid nodule, (vi) calcitonin blood level more than 100 pg/mL [20]. Moreover, there are several genetic syndromes predisposing to TC development, like multiple endocrine neoplasia type 2 (MEN2), familial adenomatous polyposis (FAP), Cowden disease, Carney complex and Werner syndrome/progeria [20,21]. Thyroid dysfunctions, especially hypothyroidism, are postulated as factors associated with increased incidence of TC [22,23]. The association between elevated body mass index (BMI) and aggressive clinicopathologic features of PTC has also been reported [24].

### 1.4. Role of Genetic Factors in Pathogenesis of TC

Progression of TC relies on both phenotypic diversity and genetic alterations. DNA sequencing studies of TC have revealed that most tumors harbor genetic aberrations affecting the mitogen-activated protein kinase (MAPK) or phosphatidylinositol 3-kinase (PI3K)/AKT signal transduction pathways [5]. These genetic alterations are predominantly point mutations in critical trigger genes of these pathways, such as BRAF, RAS or phosphatidylinositol-4,5-bisphosphate 3-kinase (PIK3CA). *RET*/*PTC* rearrangements are the other class of aberrations frequently observed in PTCs.

Pathogenesis of TC can also be associated with mutations of receptor tyrosine kinases (RTKs) and other less commonly reported pathways, including Wnt/β-catenin, FOXO3 and NF-ĸB [25,26].

**Figure 1:**
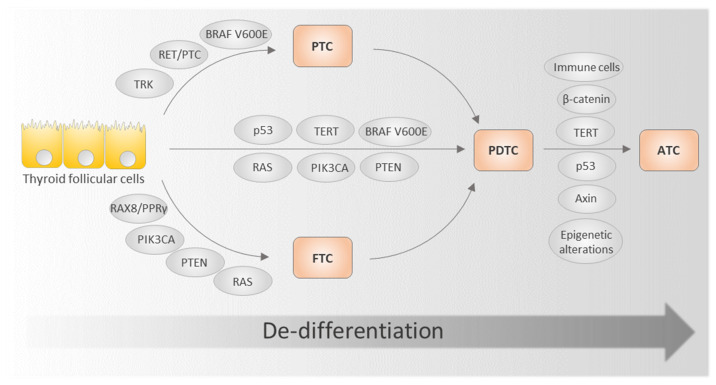
Schematic representation of TC progression showing critical genes and factors involved in de-differentiation. Modified from [29]. ATC, anaplastic thyroid carcinoma; FTC, follicular thyroid carcinoma; PDCT, poorly differentiated thyroid carcinoma; PTC, papillary thyroid carcinoma.

Genetic dysfunctions of components of these pathways, accompanied by epigenetic modifications, lead to progression of normal thyroid follicular cells to PTC or FTC [27]. The so-called “early” molecular driver alterations, *BRAF* V600E or RAS, are the most common [28]. Co-occurrence of other mutations and/or rearrangements with increased signaling of the MAPK and/or PI3K/AKT pathways likely promotes progression to PDTC [27,29,30]. Further accumulation of epigenetic and genetic alterations, as well as infiltration of the thyroid gland by immune cells, seem to be the major triggers of ATC (Figure 1) [31].

#### 1.4.1. MAPK Pathway

The MAPK pathway connects extracellular signals to intracellular transduction cascades that play a central role in regulation of cell growth. The major processes governed by the MAPK signaling chain include: proliferation, migration, apoptosis, cytoskeletal integrity, survival and cellular differentiation. In TCs, particularly in PTC, MAPK stimuli play a crucial role, as MAPK-activating mutations in genes encoding for both B-Raf proto-oncogene (BRAF) kinase and RAS kinase, or *RET/PTC* rearrangements, are frequently observed [32,33].

**Figure 2:**
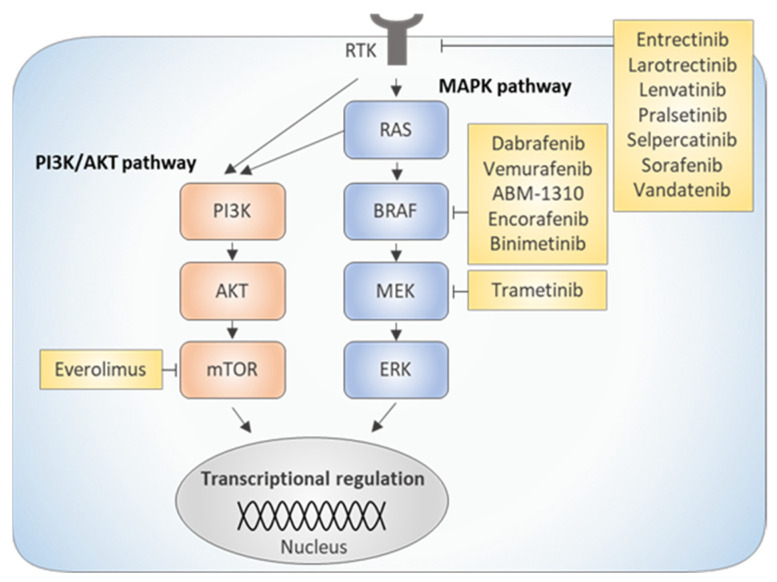
Schematic representation of major molecular targets in kinase inhibition-based therapies for thyroid cancer. RTK, receptor tyrosine kinase.

Substitution of valine with glutamic acid in codon 600 (V600E) of the *BRAF* gene encoding the BRAF serine/threonine kinase results from a thymine-to-adenine point mutation at position 1799 (T1799A). Oncogenic *BRAF* is the most frequently mutated gene in TCs and presence of the V600E alteration is reported in up to 80% of PTC cases, as well as 20–30% of ATCs [34,35,36]. The *BRAF* V600E-mutated kinase acts as the constitutive trigger of signal transmission in the MAPK pathway, independent of its upstream target, RAS, ultimately leading to increased activation of ERK kinases (Figure 2). Hyperactivation of signaling pathways, mediated by the *BRAF* V600E allele, causes impaired expression of sodium iodide symporter (NIS) and plays a key role in the radioiodine refractory (RAIR) phenomenon [37,38,39]. Some studies associate *BRAF* V600E with development of more aggressive and resistant forms of TCs [30,40]. The *BRAF* V600E allele is a useful prognostic marker determining tumor malignancy, especially in countries with a high prevalence of this genetic aberration [41,42].

#### 1.4.2. PI3K/AKT Pathway

PI3K/AKT is another signaling pathway that plays major and diverse roles in regulation of cell growth, proliferation, apoptosis, metabolism, motility, angiogenesis and resistance to treatment. Genetic alterations affecting signal transduction in this pathway involve: (i) mutations in RAS isoform-encoding genes, (ii) mutations or amplifications in the alpha catalytic subunit of PIK3CA, (iii) mutations in the *AKT* gene and (iv) mutations in the phosphatase and tensin homolog phosphatase *PTEN* [28,43]. Genetic changes in RAS family genes are considered as “early” alterations and are characteristic for DTCs, especially for FTC. However, mutations of downstream effectors of the pathway are also commonly identified in less-differentiated TCs [30,44].

The RAS family of cell membrane-anchored GTPases (HRAS, NRAS and KRAS) activates downstream proteins (e.g., PI3K and BRAF kinases) when bound to guanosine triphosphate (GTP). Constitutively activated (mutated) GTP-bound RAS isoforms stimulate downstream effector pathways, even in the absence of extracellular stimuli (Figure 2) [45,46]. The utility of *RAS* mutations as a diagnostic tool is rather low [44]. *RAS* mutations occur, on average, in 30–40% of FTCs, 30–45% of the follicular variant of PTCs (FVPTCs), in approximately 25% of ATCs and PDTCs, in 15% of HCCs and in 10–45% of MTCs. Rare cases of mutations in the classical variant of PTC have also been reported [47,48,49,50].

PTEN is a tumor suppressor that decreases the cellular levels of phosphatidylinositol 3-phosphate (PIP3) to down-regulate the activity of the PI3K/AKT pathway. PTEN loss through gene deletion, mutations and epigenetic modifications results in dysregulation of the PI3K/AKT pathway, leading to uncontrolled cell proliferation, motility and protein synthesis. PTEN alterations have been described in FTC (14%), PDTC (4%), ATC (15%), PTC (2%) and HCC (5%) [4,5,50,51,52].

Activating (missense) mutations in *PIK3CA* or copy number gains lead to increased *PIK3CA* expression. Nevertheless, its role in carcinogenesis is still not well-defined. *PIK3CA* genetic alterations are common in de-differentiated TCs, like ATCs (18%), and less frequent in FTCs (1%) and PDTCs (2%) [53,54].

The AKT kinase family is comprised of three highly homologous isoforms: AKT1, AKT2 and AKT3. Activating mutations in genes encoding these protein kinases have been detected in FTCs, PDTCs and ATCs [53,55,56]. Murine TC model-based studies have shown that AKT1 is the primary promoter of TC development and local invasion, while vascular invasion and metastatic progression are dependent predominantly on AKT1 and AKT3, and to a lesser extent, on AKT2 [57,58].

#### 1.4.3. Molecular Alterations in Receptor Tyrosine Kinases

RTKs are key regulators of cellular processes that are controlled by a variety of positive and negative feedback loops [59]. Various alterations may lead to their constant activation and triggering of signaling cascades, including MAPK and PI3K/AKT pathways, that might contribute to carcinogenesis [29,50]. The RTK family encompasses single-pass transmembrane proteins, such as RET, TRK, VEGFR and ALK. The most frequent and well-described genetic aberrations in RTK genes include rearrangements, duplications and point mutations in *RET*, *ALK* and *NTRK* [60].

*RET* is a transforming proto-oncogene. *RET* fusions or point mutations are potent oncogenic drivers in TCs. Up to a quarter of PTCs harbor *RET* fusions, where *RET/PTC1* and *RET/PTC3* rearrangements are most common. It has been reported that their prevalence is increased in patients previously exposed to high-dose radiation. *RET* fusions are also found in PDTCs and rarely in ATCs. In about 20–30% of all MTCs, germline mutations of the *RET* gene lead to hereditary MTC in the course of MEN2, whereas somatic *RET* mutations are mainly associated with sporadic MTC (approximately 50% of cases). The *RET* alteration determines distinct phenotypes of MTC that may differ in terms of age of disease onset and aggressiveness. For example, malignancies harboring the *RET* M918T somatic mutation that is the most frequent genetic change in sporadic MTCs, are related to a more aggressive MTC course and worse survival [61,62].

The anaplastic lymphoma kinase (ALK) is a transmembrane tyrosine kinase of the insulin receptor family that, upon ligand binding to its extracellular domain, promotes activation of multiple downstream signaling pathways, such as MAPK, PI3K/AKT and JAK/STAT. *ALK* gene activation, driven by mutations/rearrangements and gene fusions, is predominantly reported in PDTCs, ATCs and less frequently in PTCs and is linked with disease progression and aggressiveness [54,63,64,65].

Tropomyosin receptor kinases A, B and C (TRKA, TRKB and TRKC) are a group of receptor tyrosine kinases that play a crucial role in control and promotion of cell proliferation, survival and differentiation through the MAPK, PI3K/AKT and phospholipase C (PLC-γ) pathways. Activating DNA rearrangements that affect genes encoding TRKs (*NTRK1*, *NTRK2* and *NTRK3*) as oncogenic drivers were reported in PTCs, PDTCs and ATCs [66,67,68,69]. Recently, determination of the NTRK activation status has gained great clinical utility since the emergence of targeted inhibitor therapy, as discussed below.

#### 1.4.4. Other Molecular Alterations

PAX8-PPARγ, a nuclear transcription factor enhancing apoptosis, is the product of a gene fusion between paired box 8 (PAX8) and peroxisome proliferator activated receptor γ (PPARγ). It is suggested that the PAX8-PPARγ protein exerts a dominant negative effect on endogenous PPARγ and/or leads to the activation of subsets of PPARγ and PAX8 inducible genes. The fusion gene is detected in approximately 30% of FTCs and approximately 5% of FVPTCs, and rarely in PDTCs, HCCs and benign follicular adenoma [5,70,71,72].

*TP53* is a suppressor gene controlling the cell cycle and apoptosis, and its mutations promote tumor development and progression. *TP53* mutations are found in more than half of ATCs and in a small fraction of well-differentiated cancers [50,73,74].

The telomerase reverse transcriptase (*TERT*) gene encodes the catalytic subunit of telomerase, the enzyme responsible for adding tandem arrays of simple-sequence repeats to the ends of chromosomes and thereby preventing replicative senescence. Activating mutations in the promoter of *TERT* are believed to represent a “late” driver molecular event in the development of well-differentiated TCs. These genetic alterations are associated with tumor aggressiveness and poor outcomes in TC patients. The frequency of *TERT* mutations is 40–70% in ATCs, 40% in PDTCs, 32% in aggressive forms of HCCs, 20% in FTCs and 10% in PTCs [51,75,76]. It has been observed that enhanced activity of mutant *TERT*, which acts as an oncoprotein, accelerates further progression of cancer cells and tumor development, especially when *BRAF* V600E is already present [77]. Moreover, co-occurrence of *TERT* and *BRAF* mutations leads to failure of MAPK-targeted therapies [78].

The *EIF1AX* gene encodes for an eukaryotic translation initiation factor 1A (eIF1A), which is responsible for assembly of the ribosomal pre-initiation complex, mRNA binding, scanning and ribosomal subunit joining. Mutations in *EIF1AX* have been detected in 11% of PDCTs, 9% of ATCs and 1–2% of PTCs [5,79]. It seems that different *EIF1AX* mutations correspond to distinct phenotypes in thyroid cancer and, when they co-occur with RAS in advanced TCs, are related to higher tumor aggressiveness [80,81].

Aberrant activation of the Wnt/β-catenin signaling pathway, commonly observed during PTC initiation and progression, contributes to therapeutic resistance in cancer cells via increased expression of resistance-related genes. Among these targets of the Wnt/β-catenin signaling pathway, proteins functioning as extrusion pumps are found, such as P-glycoprotein encoded by the *ABCB1* gene [82,83]. Upregulation of the pathway confers resistance to vemurafenib, a MEK inhibitor [83]. Mutations of components of the Wnt/β-catenin signaling cascade, like β-catenin (*CTNNB1*), adenomatous polyposis coli (*APC*) or axis inhibition protein 1 (*AXIN1*), are common in less-differentiated TCs, i.e., in 60–65% of ATCs and 25% of PDTCs [82,84].

Functionally, isocitrate dehydrogenases 1 and 2 (*IDH1* and *IDH2*) play a key role within the Krebs cycle and produce α-ketoglutarate by catalyzing the oxidative decarboxylation of isocitrate. Mutations in IDH1-encoding genes are present in 16% of TCs, in particular in ATCs (11%) and FTCs (5%), whereas IDH2 mutations have been identified in only 3% of ATCs [85,86,87]. Unlike in other tumors, *IDH1* and *BRAF* (or *RAS*) mutations in TCs are not mutually exclusive. Until now, no association between the IDH mutational status and clinical characteristics has been reported [86].

## 2. Current Diagnostic and Treatment Strategies

Diagnosis of TC is required when a patient presents symptoms, like palpable thyroid lesions, enlargement of the thyroid gland (especially in a short period of time), exposure to ionizing factors in childhood or a history of thyroid disease (adenoma, goiter or thyroiditis) [12,88].

In most TC incidents it is advised to perform surgical thyroidectomy and dissection of local metastases, if present, in the shortest possible time from diagnosis. After surgery, the extent of spread of cancer is assessed using the TNM Classification of Malignant Tumors. Recommendations of surgical intervention depend on the TC type and are discussed in the next subsection. Additionally, based on the TC type, various adjuvant therapies can be applied. For ATC and MTC, complementary chemotherapy and/or radiotherapy can be performed, as well as new targeted therapies, which strictly rely on the type of molecular alterations. In patients with DTC and PDTC, the recommended adjuvant therapy is radioactive iodine/radioiodine (RAI) treatment [12,13,88].

### 2.1. Diagnosis of TC: Ultrasonography (USG) and Fine-Needle Aspiration Biopsy (FNAB)

Palpation is the first line examination of the thyroid gland, while the best tool for the diagnosis of thyroid disorders is ultrasonography examination. The frequency of diagnosed nodules in palpation is 2–6%, while USG can detect thyroid nodules even in 19–68% of randomly selected individuals [89,90]. Thyroid nodules are found in 8–65% of autopsy specimens [91]. Among the nodules detected by palpation or USG, approximately 5–15% are malignant [92,93].

USG scan is recommended as the first-line thyroid imagining technique, preceding computer tomography (CT), magnetic resonance imaging (MRI) and others. USG enables identification of features, such as shape (taller than wide), echogenity, echostructure, margin and microcalcifications, that aid differentiation between benign and malignant thyroid nodules [94]. Nevertheless, there is a need to create a pattern of thyroid cancer USG images that will enable better TC risk stratification and decrease the rate of fine-needle aspiration biopsy.

Since 2009, when Horvath published the Thyroid Imaging Reporting and Data System (TIRADS) [95], other classifications of thyroid nodules have been published, including the ATA guideline in 2015 [20], the Korean Thyroid Association (K-TIRADS) guideline in 2016 [96], the European Thyroid Association (EU-TIRADS) guideline in 2017 [97], the American College of Radiology (ACR-TIRADS) guideline in 2017 and the Chinese (C-TIRADS) guideline in 2020 [98].

Indication to FNAB is made based on the clinical and USG examination. The material obtained during FNAB is analyzed and the risk of malignancy is estimated using The Bethesda System for Reporting Thyroid Cytopathology (TBSRTC) [99]. Also, depending on the result of FNAB, the decision of surgical thyroid excision is made.

### 2.2. Surgical Treatment of TC

Although DTC patients have a more favorable prognosis than most other cancers, with an overall survival (OS) rate of 98.3% at 5 years for the majority of cases, local recurrence occurs in about 20% of cases and distant metastases are found in approximately 10% of patients [100]. PDTC has a higher risk of persistence and mortality [101,102]. According to recent guidelines [20], surgical treatment is the gold standard in TC therapy. Before cervical surgery, a detailed USG of the neck with evaluation of lymph nodes should be performed. If any suspicious lymph nodes are recognized, they should be removed simultaneously with the gland. The range of surgery depends on the type of TC and on the stratification of recurrence risk. DTC patients with low risk of recurrence can be treated with total thyroidectomy or lobectomy with isthmusectomy. DTC and PDTC patients with intermediate or high risk of recurrent disease undergo total thyroidectomy and therapeutic neck dissection with or without prophylactic central neck dissection. Patients with intermediate or high risk of recurrence or with distant metastases should be treated with radioiodine [13].

ATC is an undifferentiated subtype of TCs that is resistant to therapy with the iodine-131 radioisotope. Due to an inauspicious prognosis, TNM classification for ATC only provides stage IV, which is subdivided into three classes: IVA, IVB and IVC. Surgery, followed by chemotherapy and radiotherapy, is recommended as the first choice of intervention for management of ATC, provided that a full resection of the tumor can be obtained and no distant metastases are identified (stage IVA). Patients with unresectable ATCs or with local invasion and small-volume metastases confirmed in the neck (IVB) should be treated with radiation therapy and adjuvant chemotherapy, without surgical intervention. Furthermore, systemic therapies with or without palliative radiotherapy should be considered for patients with large-volume or distant metastases. Recent ATA Guidelines for Management of Patients with ATC, which were released in March 2021, incorporate targeted therapies with kinase inhibitors in their recommendations [103], as discussed below.

Patients with MTC have poor prognosis, with a 10-year survival rate at approximately 50% and distant metastases observed in 7–23% of cases at the moment of recognition. The first-choice treatment is surgical thyroidectomy and central compartment neck dissection, but it is curative only for localized MTCs. In case of widespread regional or metastatic disease, surgical interventions are not associated with a higher cure rate, therefore, less aggressive local treatment procedures should be preferred. Systemic therapy is employed in multi-metastatic and rapidly progressing malignancies [61,104,105].

### 2.3. RAI Treatment

The thyroid gland has the unique ability to uptake and concentrate iodide, which is a critical step in biosynthesis of TH. Iodide is actively transported from the blood through the basolateral plasma membrane by the NIS protein, whereas the anion transporter pendrin and other proteins mediate iodide efflux to the follicular lumen. Thyroid peroxidase (TPO) attaches oxidized iodide to tyrosine residues of thyroglobulin (Tg) producing monoiodotyrosine (MIT) and diiodotyrosine (DIT). Subsequently, TPO couples a MIT and a DIT residue to form triiodothyronine (T3) or two DIT residues to form thyroxine (T4) [1,106].

The ability to uptake the iodine-131 radioisotope is preserved in most cases of DTC, therefore RAI has become a basic treatment modality in DTC. The accumulated radioiodine predominantly emits β-particles, which penetrate a short distance of only a few millimeters causing death of cancer cells. RAI is recommended as remnant ablation for patients with intermediate-risk DTC and as an adjuvant therapy for patients with high-risk DTC. For patients with low-risk DTC, who have undergone less than total thyroidectomy, RAI is not recommended [13,20,107].

De-differentiated TCs, like ATC and some PDTCs, lose their functional ability to transport and accumulate iodine due to several alterations. The same phenomenon occurs in 5–15% of DTC incidents [108], while approximately 50% of metastatic DTCs are refractory to RAI treatment [109,110].

DTCs that are metastatic or locally advanced and have one or more metastatic sites without any radioiodine uptake, or for which significant progression of the disease is observed during the year after radioiodine treatment, are considered as RAIR. RAIR-DTC significantly reduces the mean life expectancy by 3–5 years [111].

The other molecular mechanism harming iodine uptake is an underpinning disfunction of NIS, which occurs predominantly in *BRAF*-mutated tissue. However, other aberrant activations of MAPK and PI3K/AKT pathways are also associated with this phenomenon. Much effort has been made to identify small molecule inhibitors that would be able to restore and enhance NIS protein expression and its physiological functions [112,113]. It seems that BRAF/MEK inhibitors are the most promising agents for selected DTCs, nevertheless, prospective multicenter trials are required to evaluate the efficacy and safety of iodine uptake restoration on a larger cohort of patients [37,39].

## 3. Currently Used and Investigated Targeted Therapies in TCs

Although most TC cases can be cured by combined surgical and RAI treatment, all ATCs and MTCs, as well as some DTC incidents, require further therapy. Standard chemotherapy, if recommended, is used in ATC treatment, whereas in DTC, only doxorubicin has been approved for RAIR-DTC patients. Further treatment of MTC relies on removal of metastases (e.g., external beam radiation therapy, EBRT) and no systemic chemotherapy is recommended. Due to numerous adverse events and lack of survival rate enhancement, standard chemotherapy is insufficient in treatment of TCs. Over the last decade, knowledge about the molecular basis of TC carcinogenesis has led to the development of kinase inhibition-based therapies, which allow for targeting of tumor cells and are thus considered as a novel tool for personalized medicine [43].

Kinase inhibitors (KIs) are small molecular compounds. Most of the Food and Drug Administration (FDA)-approved inhibitors for DTC, ATC and MTC competitively block the ATP-binding pocket of protein kinase(s), thereby inhibiting cell signaling and proliferation. First generation KIs, such as lenvatinib, sorafenib, vandetanib and cabozantinib, present a broad-spectrum of activity and are known as multi-kinase inhibitors (MKIs). Due to their low selectivity, MKIs have more off-target side effects leading to certain adverse events (AEs). The higher selectivity of the second generation of specific kinase inhibitors (SKIs) that target individual protein kinases, e.g., BRAF-specific inhibitors, makes those drugs more tolerable and less AEs-triggering [114,115,116].

Ten years have passed since the first targeted kinase inhibitor, vandetanib, was approved and registered for treatment of symptomatic or progressive MTCs. Since then, other MKIs, such as sorafenib, lenvatinib, vandetanib and cabozantinib, were approved for TC treatment by both the FDA and the European Medical Agency (EMA). Lenvatinib and sorafenib are used for treatment of advanced RAIR-DTC, while vandetanib and cabozantinib are administered in MTC therapy. Apart from the approved indications, MKIs are used in other subtypes of TCs with variable effect [28,114,115].

Larotrectinib, entrectinib and selpercatinib represent selective FDA- and EMA-approved inhibitors. Pralsetinib (BLU-667), a selective inhibitor targeting mutated and fused forms of RET, has been approved by the FDA and is under evaluation by the EMA [115]. Combinatory therapy using dabrafenib and trametinib, which are selective inhibitors of MEK and BRAF, respectively, has been approved by the FDA for ATC treatment in patients with locally advanced or metastasized disease [103]. Kinase inhibitors that have been approved for TC therapy are summarized in Table 1. The clinical and preclinical approaches from the last two years regarding the use of kinase inhibitors in the treatment of TCs are discussed below.

### 3.1. Targeted Therapies for DTC Treatment

Guidelines for DTC treatment that were developed in 2015 [20], include recommendations for KI usage in metastatic, rapidly progressive and symptomatic RAIR-DTC cases. A variety of novel agents and combinatory treatments are being tested as candidate clinical approaches in DTC and progressive metastatic RAIR-DTC (summarized in Table 2). Special attention is paid to inhibitors directed against BRAF, RET, MEK and TRK kinases, which will “re-sensitize” DTC and RAI-refractory tumors.

#### 3.1.1. MKI-Based Therapies of DTC

Recent studies on the use of MKIs in RAIR-DTCs showed that treatment with lenvatinib or sorafenib results in an improvement of progression-free survival (PFS) [117], objective tumor response rate and OS. However, during treatment, dose modifications are required to reduce AEs [118]. A long-term evaluation of apatinib-based therapy in a phase II trial for progressive RAIR-DTC displayed sustainable efficacy and a tolerable safety profile, therefore, it can be considered as a promising treatment option [119]. The benefit from apatinib therapy may result from the effective inhibition of proliferation and migration of PTC cells, as well as from induction of apoptosis, cell cycle arrest and autophagy, as was recently demonstrated in an in vitro study [120]. Donafenib tested in a randomized, open-label, multicenter phase II trial of progressive locally advanced or metastatic RAIR-DTC, demonstrated improvement in PFS, partial response (PR) and the overall response rate (ORR). That effect was dose-dependent and effective for both used regimens, but a higher rate of AEs was reported in the higher dose group [121]. In a randomized, double-blind, placebo-controlled phase III trial, treatment with cabozantinib (an agent already approved for MTC therapy) prolonged PFS in RAIR-DTC patients, but at the same time accounted for a relatively high rate of AEs [122].

The results of Real-World Studies (RWS) showed that treatment with lenvatinib, sorafenib and pazopanib, followed or accompanied by another therapy, may improve the clinical outcome (i.e., higher PFS) of patients with RAIR-DTC [123,124,125]. However, the observed AEs, most frequently fatigue, asthenia and hypertension (HT), forced to reduce the MKI dose. Despite that, a dose reduction likely did not abrogate the apparent efficacy of therapy [126]. A recently published report, comparing sorafenib and lenvatinib for RAIR-DTC treatment, showed that in the group treated with lenvatinib, PR to treatment was significantly increased, and was accompanied by a drop in serum Tg levels [127]. Even though MKIs are considered as a promising treatment for RAIR-DTC, the use of them should be cautious due to a high frequency of AEs [128].

There is increasing interest in MKI-based therapies in combination with immunotherapy (e.g., NCT04560127, NCT03732495), MAPK- or PI3K/AKT-SKIs (e.g., NCT02143726, NCT01947023) or radioiodine (NCT04952493). In the recently reported preliminary studies on a small, randomized, multi-institutional phase II study (NCT02143726), where everolimus (mTOR inhibitor) and sorafenib co-therapy was applied for patients with RAI refractory HCC, improved PFS was observed [129]. Similarly, in a preclinical study, DTC cells treated with a combined therapy, including lenvatinib and radiation, synergistically induced apoptosis and G2/M phase arrest, as well as inhibited colony formation in vitro (cell lines) and tumor growth in nude mice [130].

#### 3.1.2. Single Kinase-Targeted Therapies of DTC

Since MAPK activation, mediated by a *BRAF* single-nucleotide mutation, is one of the most critical events in DTC development, there are ongoing efforts to investigate BRAF-specific SKIs either in monotherapy or in combination with other agents. For example, a recent in vitro study performed on *BRAF* V600E-positive TC cell lines indicated antiproliferative and re-differentiative effects of dabrafenib and vemurafenib [131]. Moreover, vemurafenib-treated *BRAF* V600E-positive PTC cells exhibited an increased apoptosis level [132], whereas ascorbic acid (vitamin C) was found to sensitize *BRAF* V600E-positive thyroid cancer cells to this agent [133]. Recent reports suggest, that co-therapy based on dabrafenib and trametinib may lead to durable disease control and prolonged benefit in patients with a *BRAF*-mutated PTC [134]. This combination of drugs is currently under investigation in a panel of clinical trials, such as NCT03244956, NCT04619316 and NCT04554680 (Table 2). Simultaneous treatment with BRAF and MEK inhibitors upregulates NIS expression, suggesting it may improve RAI responsiveness [135]. According to preclinical data, this effect may be amplified when either dabrafenib or vemurafenib is used in combination with follistatin or vactosertib, SMAD pathway inhibitors [136]. A prospective, multicentric, open-label phase II trial (MERAIODE, NCT03244956) evaluating the efficacy and tolerance of trametinib and dabrafenib treatment followed by administration of RAI in patients with metastatic DTC provided promising results. The therapy restored radioiodine accumulation in *BRAF*-mutated patients and led to tumor control in 90% of cases [137].

In a phase I, open label study of lifirafenib (BGB-283), a RAF family kinase inhibitor targeting *BRAF* V600E, EGFR and RAS proteins, four patients with *BRAF* V600E-positive TCs were enrolled, including one in the dose-escalation phase and three in the dose-expansion phase. Lifirafenib demonstrated antitumor activity with PR and an acceptable risk benefit profile in *BRAF* V600E-positive solid tumors [138]. Recently, enrollment for a phase I/II trial for assessment of the safety and efficacy of lifirafenib in combination with a MEK inhibitor (mirdametinib) in patients with *BRAF*- and *RAS*-mutated tumors was commenced (NCT03905148).

The utility of therapies based on inhibitors specific for tropomyosin receptor kinases in *NTRK* gene fusion-positive solid tumors was also investigated. Entrectinib (RXDX-101), a potent inhibitor of TRKA, TRKB and TRKC, was reported to induce durable and clinically meaningful responses in patients with *NTRK* fusion-positive solid tumors [139]. A phase I study recruitment of patients with PTC (STARTRK-1, NCT02097810) was completed in June 2021 and a phase II trial (STARTRK-2, NCT02568267) is still recruiting. Another TRK inhibitor, larotrectinib, was found to restore radioiodine avidity in PTC-pediatric patients, leading to inhibition of tumor growth [140].

The common activating *RET* alterations in PTC cases encouraged researchers to use RET kinase inhibitors for PTC therapy. The results of selpercatinib treatment in two independent case reports of patients with *RET*-altered PTC (*NCOA4-RET* and *CCDC6-RET* fusions) showed restored radioiodine avidity and a re-differentiation effect suppressing Tg after six months of treatment [140,141].

A report on ALK-targeted therapy in RAIR-PTC patients harboring an *EML4-ALK* gene fusion variant 3 has just been released. In the experimental approach, the authors established a cell line derived from a PTC patient to select the most potent ALK-specific inhibitor in vitro. Tumor cell line data showed that lorlatinib (3rd generation ALK TKI) was a more potent drug than the previously administered crizotinib (1st generation ALK TKI). After lorlatinib therapy, the patient exhibited a significant decrease in Tg levels, a partial drop in PR, with a decrease in the sum of the target lesions [142].

### 3.2. Targeted Therapies for ATC Treatment

Recent ATA guidelines for management of ATC patients with grade IVB and IVC recommends mutation-guided individualized targeted therapeutic strategies based on KIs. Therefore, extended molecular profiling of ATC cases is strongly recommended, as it may reveal promising possibilities for target-specific therapies [143]. If it is possible, patient participation in clinical trials should be taken under consideration [103]. The list of on-going clinical trials for treatment of ATC is provided in Table 3.

#### 3.2.1. MKI-Based Therapies of ATC

Over the last two years, a few studies have reported the results of MKI-based approaches in ATC treatment. Lenvatinib, an oral multitarget inhibitor, has been used in adjuvant therapy after thyroidectomy with neck dissection and postoperative chemoradiotherapy. Therapy extended the time to progression, but some AEs, like HT fatigue, anorexia and severe drug-induced hepatitis, forced investigators to taper the drug dose [144,145]. Results of treatment of ATC patients with lenvatinib (phase II trial) confirmed tumor size reduction (in more than half of evaluated patients), PR to treatment (*n* = 1) and >30% reduction in the total size of the target lesion. Nevertheless, lack of efficacy per prespecified criteria in interim analysis led to end the study at the enrolment stage. The authors suggested that lenvatinib, when used alone, may not be an effective treatment for ATC [146]. Another preclinical study demonstrated that combination of lenvatinib with the MEK inhibitor selumetinib (AZD6244) enhanced the antitumor effects of monotherapy in vitro and in an ATC mouse model. These effects may occur through the PI3K/AKT and MAPK signaling pathways [147]. Sorafenib, an oral MKI, exerts activity against ATC cells, especially in combination with other drugs. Simultaneous administration of sorafenib and an agent restoring molecular function of the p53 protein (CP-31398) was reported to decrease viability of ATC-derived cells (SW579) [148]. Another study showed that combination of *N*-hydroxy-7-(2-naphthylthio) heptanamide (HNHA), sorafenib and radiation was effective in inducing apoptosis and cell cycle arrest, leading to significant suppression of tumor growth in a mouse xenograft model and may be considered as a potential approach to ATC treatment [149].

#### 3.2.2. Single Kinase-Targeted Therapies of ATC

Since AEs are a significant limitation of the currently available MKI-based therapies, identification of novel molecule inhibitors for ATC management is needed. CTOM-DHP is a promising SKI specifically inhibiting both MAPK and PI3K/AKT signaling pathways. Administration of this compound to the ATC-originated cell line and tumor xenograft mice model led to increased NIS promotor expression, RAI avidity (both I^124^ and I^131^ uptake) and cytotoxicity. Therefore, CTOM-DHP may be considered as a potent compound for restoring RAI avidity in ATC [112]. Furthermore, it is postulated that combinatory approaches, rather than single inhibitor-based therapies, may provide more profits for patients. As already mentioned above, co-administration of selective BRAF and MEK inhibitors (dabrafenib and trametinib, respectively) has received FDA approval for treatment of locally advanced and metastatic *BRAF* V600E-positive ATCs [103]. A retrospective study on such combinatory treatment showed prolonged survival of *BRAF*-mutant ATC patients [150]. Additionally, a recently released case report demonstrated a good rapid response in a patient treated with dabrafenib and trametinib who had already received other BRAF-targeted inhibitors [151]. Moreover, co-administration of BRAF-directed agents with other drugs, like check point inhibitors (pembrolizumab), may prolong survival of patients [152]. In in vitro studies, exposure of *BRAF* V600E-positive ATC cell lines to dabrafenib and melatonin showed synergistic inhibition of hTERT, and in consequence, cell arrest in the G1 phase and decreased cell viability [153]. Similarly, usage of another SKI, vemurafenib with a STAT3 pathway inhibitor on ATC-derived cell lines (sphere and monolayer) and mouse xenografts resulted in decreased viability and increased rate of cell apoptosis. These results indicate that inhibition of other pathways, like STAT3, could enhance the sensitivity of kinase inhibitors in ATC cells [154].

### 3.3. Targeted Therapies for MTC Treatment

Due to their origins, MTCs are unable to accumulate radioiodine, thus only surgical- and systemic-based treatments may be applicable in clinical practice. Most MTCs occur sporadically, but in approximately one-fifth of cases, they are familial and caused by germline mutations of the *RET* proto-oncogene. Somatic *RET* mutations are found in approximately half of patients with sporadic MTCs. *RAS* gene mutations, and less often *ALK* fusions, are alternative genetic events spotted in sporadic MTCs. Apart from these alterations of the proto-oncogene, overexpression of vascular endothelial growth factor (VEGF) receptors is often detected in the disease. The advent of targeted small-molecule KIs has revolutionized medical treatment of MTCs. As already mentioned, current guidelines on management of MTC patients recommend cabozantinib and vandetanib as the first-line single-agent systemic therapy in patients with advanced progressive MTC, based on their documented ability to improve PFS (Table 1). Both drugs inhibit RET kinase activity to some extent, however, their major anticancer effect is due to their strong inhibition of angiogenesis [13,61,105,155]. The list of on-going clinical trials for treatment of MTC is provided in Table 4.

#### 3.3.1. MKI-Based Therapies of MTC

In 2021, the results of a comprehensive retrospective study, which aimed to identify whether approval of MKIs for MTC is associated with changes in systemic therapy administration or changes in overall survival, was published [156]. The clinicopathologic comparisons were conducted between pre-multikinase (2005–2010) and post-multikinase inhibitor (2011–2016) approval groups. A total of 2891 patients were enrolled, including 1265 cases in the pre-MKI and 1626 cases in the post-MKI approval group. The results showed that after approval of MKIs, the rate of systemic treatment administration significantly increased (8.3% and 11.3% in pre-MKI and post-MKI, respectively). Nevertheless, no improvements in OS were detected between those two groups of patients. In 2020, a post hoc analysis phase III trial (ZETA trial) involving patients with advanced MTCs treated with vandetanib demonstrated increased PFS and improved ORR compared to a placebo; however, OS did not significantly differ between vandetanib- and placebo-treated patients. In contrast, time to worsening of pain, which was predefined as an endpoint to assess symptomatic benefits of the trial, was found to be extended in the vandetanib group [157]. According to recent reports [158,159], one in four patients treated with vandetanib or cabozantinib developed AEs, which required withdrawal of the drug, or exhibited drug resistance. Therefore, extensive efforts are made to broaden the panel of MKI-based therapies for MTC patients. For example, Matrone et al. (2021) evaluated the impact of the off-label use of lenvatinib in a group of ten patients affected by locally advanced, non-resectable, metastatic MTCs with previous failure of other TKIs [160]. Lenvatinib induced substantial stabilization of metastatic lesions and disease control. The observed AEs were consistent with other studies and could be managed by personalization of the initial dose and one or more prompt dose reductions. It seems that this agent could be effective as salvage therapy in cases, where no other treatment strategies are available. Moreover, due to the absence of cross-resistance between vandetanib and lenvatinib, second- and third-line kinase inhibitor treatment should always be considered [160]. A multicenter, randomized, double-blind, placebo-controlled phase IIB study investigated the usefulness of a novel MKI, anlotinib, in the therapy of locally advanced or metastatic MTCs. Drug treatment, compared to placebo, significantly improved the median PFS and ORR, with no significant difference in OS between the tested groups [161].

#### 3.3.2. Single Kinase-Targeted Therapies of MTC

Since the release of the MTC management guidelines [105] in 2020, the FDA has approved two new, highly potent RET-selective tyrosine kinase inhibitors, pralsetinib and selpercatinib. Both agents are destined for individuals 12 years or older with advanced, metastatic *RET*-mutant MTCs or other *RET* fusion-positive TCs, which require systemic therapy. Selpercatinib has also been approved by the EMA for patients aged ≥ 12 years, who were previously treated with cabozantinib and/or vandetanib, while pralsetinib is still awaiting a positive opinion. Pralsetinib and selpercatinib recommendations were based on multicenter, open label, multi-cohort clinical trials, ARROW, NCT03037385 [162] and LIBRETTO-001, NCT03157128, respectively, that investigated their efficacy in MTC patients with *RET* gene alterations. These potent inhibitors not only improved ORR, but also higher PFS and OS, with a lower rate of AEs was observed [162,163]. Unfortunately, in a number of cases, there were no improvements after treatment and resistance-associated mutations were identified [62,163,164]. Several recent studies have shown that both pralsetinib and selpercatinib are inefficient in MTC patients with *RET* non-gate mutations at the front and at the hinge of the receptor. However, strong pralsetinib-resistant L730V/I mutations, located at the roof of the solvent front of the RET ATP-binding site, remained sensitive to selpercatinib in the preclinical study [165]. These findings highlight the need to develop a next-generation of drugs covering both gatekeeper and non-gatekeeper mutations for on-target resistance, in addition to deciphering patterns of off-target resistance by alternative mechanisms for combinatory therapies [62,164].

## 4. Treatment-Related Toxicities

MKI-related toxicities (listed in Table 5) are very common and likely reflect target-binding affinities specific to each drug [62,166,167,168,169]. Among the AEs observed during treatment, HT appears to be the most frequent and is managed using standard antihypertensive drugs. Early diagnosis of HT may help avoid serious complications and prevent premature termination of MKI-based therapies in patients [38,168]. Administration of MKIs is also frequently associated with an increased risk of palmar-plantar erythrodysesthesia syndrome (PPES), characterized by tingling and tenderness, with more serious symptoms including symmetrical redness, swelling and pain on the palms and soles. The etiology of PPES is still unclear and its management is predominantly symptomatic with dose reduction or interruption for severe cases. Other reported muco-cutaneous AEs, such as rash, alopecia and oral stomatitis, are specifically related to inhibition of the VEGFR/EGFR pathway [162,168]. Proteinuria is a common renal side effect of antiangiogenic MKIs [166]. All MKI treatments evaluated in TCs are related to increased risk of gastrointestinal toxicities, including nausea, vomiting, diarrhoea, mucositis, weight loss and hepatic impairment [122,166]. Fatigue, also defined as asthenia, can arise as a common AE during treatment with MKIs [168]. Recently, Monti et al. (2021) suggested that adrenal insufficiency may be responsible for lenvatinib-associated fatigue (especially in patients experiencing extreme fatigue) [170]. Preliminary findings suggest that early diagnosis of primary adrenal insufficiency is important, since cortisone acetate replacement therapy can improve fatigue in patients without the need of dose reduction of MKIs [170]. Thyroid function and thyroid hormone metabolism are the most commonly reported endocrine toxicities caused by MKIs, especially those targeting VEGFR [171]. The majority of severe side effects occur early in the course of treatment, and if managed, patients may experience persistent long-term disease control [172]. Side effects occur more frequently in older patients and may differ according to the patient’s ethnicity [169].

The most frequent AEs in BRAF- and MEK-targeted therapies are fatigue, fever, diarrhoea, HT and hyperproliferative cutaneous events [38,173]. Most are grade 1 and 2 and are consistent with those reported for BRAF or MEK inhibitors alone [173]. The incidence of AEs in combinatory therapy with dabrafenib and trametinib is highest during the initial six months of treatment and declines thereafter [173]. These findings emphasize the need for proper management of AEs during early treatment, similarly to MKI-based therapies, to avoid poor adherence and early discontinuation from the treatment [166,167,173]. RET-targeted therapies, based on selpercatinib and pralsetinib, seem to exhibit lower side effects than other FDA-approved treatments (Table 5). The majority of patients tolerate these drugs well, while only a few percent of patients discontinue the therapy due to treatment-related AEs [62,162,163]. The most commonly observed AEs of selpercatinib are HT, diarrhoea, fatigue and dry mouth [163], whereas pralsetinib administration is associated with increased risk of neutropenia and liver impairment as well as decreased white blood cells (WBCs) count [162]. Overall, comprehensive education of patients to increase their awareness of signs and symptoms of possible side effects and continued monitoring is critical.

## 5. Conclusions and Future Perspectives

Novel tyrosine kinase inhibitor-based approaches have been studied, alone or in combination, to improve the inauspicious prognosis of TC patients. The results of recent studies highlight new treatment opportunities for RAIR-DTC, ATC and MTC patients. Prolonged progression-free and overall survival are possible with targeted therapeutic agents. Prospectively, information on molecular aberrations will be more important than the histological type of TC and will be crucial for clinical decision-making.

Implementation of kinase inhibitors promotes approaches, which are related to the individual needs of patients. Such personalized, low-risk therapies, in which the administrated selective KIs target certain specific aberrations, are possible due to the increasing availability of advanced molecular tests that allow for rapid mutational analysis of TCs. Moreover, therapy of TCs will be based upon selection of chemotherapeutics previously used in other cancer types, while the aim of treatment will focus on the point of action, i.e., place and type of mutation.

Despite remarkable progress, there continue to be two broad categories of barriers in clinical usage of targeted therapeutics in TCs, i.e., limiting adverse effects and development of resistance. Targeted agents usually have less severe and long-term AEs than conventional chemotherapy. However, they should be considered as chronic approaches, and appropriately managed to prolong treatment duration in patients exhibiting a clinical benefit [38]. The aquired drug-resistance phenomenon, which results from systemic therapies, remains a concern. For example, up to half of patients treated with BRAF-targeted SKIs eventually develop resistance. It is typically mediated through reactivation of the MAPK pathway and can occur through several mechanisms, including upstream activating mutations (e.g., mutations in RAS-encoding genes), downstream MAPK pathway alterations (e.g., *MEK* and *ERK* mutations), activation of parallel signaling pathways (e.g., PI3K/AKT), increase in the expression level of RTKs (e.g., EGFR) and *BRAF* gene amplification and alternative splicing. The failure of targeted therapies could also result from overexpression of ATP binding cassette (ABC) drug efflux pumps [173,174]. Recent studies demonstrated that overexpression of *ABCB1* is sufficient to confer resistance of cancer cells to kinase inhibitors [175,176]. The phenomenon of multidrug resistance requires use of combinatory therapies that are composed of drugs with differing molecular targets, mechanisms of action and various signaling pathways. Thus, both non-selective and selective kinase inhibitors, as well as immunomodulatory drugs, check-point inhibitors and agents that influence other signaling pathways will be implemented in management and therapy of TCs.

## Figures and Tables

**Table 1:** Kinase inhibitors currently approved for thyroid cancer (TC) treatment.

Cancer Type	Agent (Brand Name(s))	Time of Approval	Point of Action	Indication
DTC				
	Entrectinib (Rozlytrek)	FDA 2019 EMA 2020	*NTRK*	Solid tumors with a *NTRK* gene fusion without a drug-resistance mutation in certain TRK proteins
	Larotrectinib (Vitrakvi)	FDA 2018 EMA 2019	*NTRK*	Solid tumors with a *NTRK* gene fusion without a drug-resistance mutation in certain TRK proteins
	Lenvatinib (Lenvima)	FDA 2015 EMA 2014	MKI	Progressive, recurrent or metastatic RAIR-DTC
	Pralsetinib (Gavreto)	FDA 2020	*RET*	Metastatic or advanced TCs with a *RET* fusion gene
	Selpercatinib (Retevmo, Retsevmo)	FDA 2020 EMA 2021	*RET*	FDA: metastatic or advanced RAIR-DTC patients aged ≥ 12 years with a *RET* fusion gene; EMA: advanced DTC patients with changes in *RET* gene previously treated with sorafenib and/or lenvatinib
	Cabozantinib (Cabometyx, Cometriq)	FDA 2021	MKI	Advanced or metastatic RAIR-DTC that has progressed following prior VEGFR-targeted therapy
	Sorafenib (Nexavar)	FDA 2005 EMA 2006	MKI	Progressive, recurrent or metastatic RAIR-TC
ATC				
	Dabrafenib (Tafinlar)	FDA 2018	*BRAF*	Locally advanced or metastasized ATC that cannot receive local treatment; Therapy in combination with trametinib
	Entrectinib (Rozlytrek)	FDA 2019 EMA 2020	*NTRK*	Solid tumors with a *NTRK* gene fusion without a drug-resistance mutation in certain TRK proteins
	Larotrectinib (Vitrakvi)	FDA 2018 EMA 2019	*NTRK*	Solid tumors with a *NTRK* gene fusion without a drug-resistance mutation in certain TRK proteins
	Pralsetinib (Gavreto)	FDA 2020	*RET*	Metastatic or advanced TCs with a *RET* fusion gene
	Selpercatinib (Retevmo, Retsevmo)	FDA 2020 EMA 2021	*RET*	FDA: metastatic or advanced ATC patients aged ≥ 12 years with a *RET* fusion gene; EMA: advanced ATC patients with changes in *RET* gene previously treated with sorafenib and/or lenvatinib
	Trametinib (Mekinist)	FDA 2018	*MEK*	Locally advanced or metastasized ATC that cannot receive local treatment; Therapy in combination with dabrafenib
MTC				
	Cabozantinib (Cabometyx, Cometriq)	FDA 2012 EMA 2014	MKI	Progressive or metastasized MTC
	Pralsetinib (Gavreto)	FDA 2020	*RET*	Advanced or metastatic MTC with a mutation in the *RET* gene
	Selpercatinib (Retevmo, Retsevmo)	FDA 2020 EMA 2021	*RET*	FDA: advanced or metastatic MTC aged ≥ 12 years with a mutation in the *RET* gene; EMA: advanced MTC patients aged ≥ 12 years with changes in *RET* gene previously treated with cabozantinib and/or vandetanib
	Vandetanib (Caprelsa)	FDA 2011 EMA 2013	MKI	Locally advanced or metastasized MTC

ATC, anaplastic thyroid carcinoma; DTC, differentiated thyroid carcinoma; EMA, European Medical Agency; FDA, Food and Drug Administration; MKI, multikinase inhibitor; MTC, medullary thyroid carcinoma; RAIR, radioiodine refractory.

**Table 2:** Kinase inhibitors currently investigated in clinical trials for DTC treatment (registered under ClinicalTrials.gov and clinicaltrialsregister.eu).

Agent (Drug Name)	Alternative/Control Treatment	Study Phase	Specific Inclusion Criteria	Study Start/ Estimated Completion Date	Status	Trial Number
MKIs						
Anlotinib	ND	II	ND	01.12.2020/ 07.12.2022	Recruiting	NCT05007093
Apatinib	ND	II	*VEGFR2*	10.04.2019/ 30.05.2020	Recruiting	NCT04180007 (APT-01)
Apatinib	Placebo	III	*VEGFR2*	12.2016/ 06.2022	Active, not recruiting	NCT03048877
Cabozantinib	ND	II	ND	01.2014/ 05.2022	Active, not recruiting	NCT02041260
Cabozantinib	Placebo	III	ND	05.10.2018/ 12.2022	Active, not recruiting	NCT03690388
Donafenib	Placebo	III	ND	29.04.2018/ 12.2021	Recruiting	NCT03602495
Lenvatinib	ND	I/II	FTC, PTC	29.12.2014/ 31.03.2022	Active, not recruiting	NCT02432274 (in Cohort 2A)
Lenvatinib	ND	II	ND	01.05.2021/ 01.01.2024	Not yet recruiting	NCT04858867
Lenvatinib	ND	III	ND	11.01.2017/ 30.10.2021	Active, not recruiting	NCT02966093
Entrectinib	ND	II	*NTRK*, *ROS1* or *ALK* PTC	01.12.2022/ 02.12.2024	Recruiting	NCT02568267 (STARTRK-2)
Imatinib	ND	I	PTC	18.09.2018/ 30.12.2021	Recruiting	NCT03469011
Vandetanib	Placebo	II	FTC, PTC	29.09.2007/ 12.2021	Active, not recruiting	NCT00537095
Vandetanib	Placebo	III	ND	17.09.2013/ 31.12.2021	Active, not recruiting	NCT01876784
TRK inhibitors						
Larotrectinib	ND	II	*NTRK*	30.09.2015/ 30.09.2025	Recruiting	NCT02576431 (NAVIGATE)
ALK inhibitors						
Alectinib	ND	II	*ALK*-mutant PTC	24.05.2021/ 01.12.2025	Recruiting	NCT04644315
Repotrectinib	ND	I	*ALK*, *ROS1* or *NTRK* rearrangements	27.02.2017/ 12.2022	Recruiting	NCT03093116 (TRIDENT-1)
MEK and BRAF inhibitors (combination therapies)						
Cobimetinib/ ABM-1310	ND	I	*BRAF* V600E	16.06.2020/ 12.2021	Recruiting	NCT04190628
Encorafenib/ Binimetinib	Encorafenib/ Binimetinib/ Nivolumab ^1^	II	*BRAF* V600E/M	30.10.2020/ 01.08.2024	Recruiting	NCT04061980
Trametinib/I^131^	ND	II	*RAS*-mutant or *RAS*/*BRAF* wt	14.08.2014/ 31.12.2021	Active, not recruiting	NCT02152995
Dabrafenib/ Trametinib	ND	II	*BRAF* V600E or *RAS*	27.12.2017/ 12.2022	Recruiting	NCT03244956 (MERAIODE)
Trametinib	Trametinib/ Dabrafenib	II	*BRAF* wt (Trametinib) and *BRAF* V600E (Trametinib/ Dabrafenib)	05.02.2018/ 30.06.2022	Recruiting	NCT04619316 (ERRITI)
Dabrafenib/ Trametinib	ND	II	*BRAF* V600E or *RAS*	30.12.2020/ 04.2022	Recruiting	NCT04554680
Trametinib	Trametinib/ Dabrafenib	I (pilot study)	*BRAF* wt (Trametinib) and *BRAF* V600E (Trametinib/ Dabrafenib) RAIR-PTC	ND	Ongoing	2016-002941-4 (ERRITI)
Dabrafenib/ Trametinib	Placebo	III	*BRAF* V600E	01.10.2021/ 30.10.2026	Not yet recruiting	NCT04940052
Vemurafenib	ND	II	PTC	07.11.2012/ 30.11.2020	Active, not recruiting	NCT01709292
Other combination therapies						
Anlotinib/I^131^	ND	II	ND	15.08.2021/ 20.07.2024	Not yet recruiting	NCT04952493
Apatinib/ Camrelizumab ^1^	ND	II	ND	23.09.2020/ 30.06.2023	Recruiting	NCT04560127
Cabozantinib/ Nivolumab ^1^/ Ipilimumab ^2^	ND	II	ND	15.07.2019/ 15.01.2022	Recruiting	NCT03914300
Cabozantinib/ Atezolizumab ^1^	ND	I	ND	05.09.2017/ 12.2022	Recruiting	NCT03170960
Lenvatinib/ Pembrolizumab ^1^	ND	II	ND	08.10.2021/ 30.09.2022	Recruiting	NCT02973997
Lapatinib/ Dabrafenib	ND	I	*BRAF*	29.08.2013/ 01.07.2022	Active, not recruiting	NCT01947023
Lenvatinib/ Denosumab ^3^	ND	II	ND	26.07.2019/ 15.06.2022	Recruiting	NCT03732495 (LENVOS)
Sorafenib/ Everolimus ^4^	ND	II	TC	06.2010/ 06.2022	Active, not recruiting	NCT01141309
Sorafenib/ Everolimus ^4^	ND	II	DTC progressed on monotherapy with Sorafenib	10.2010/ 03.2021	Active, not recruiting	NCT01263951
Regorafenib/ Avelumab ^1^	ND	I/II	ND	04.05.2018/ 05.2022	Recruiting	NCT03475953 (REGOMUNE)
Sorafenib/ Everolimus ^4^	Sorafenib alone	II	RAIR-HCC	01.10.2014/ 28.01.2021	Active, not recruiting	NCT02143726
Selumetinib	Selumetinib/I^131^	II	ND	04.05.2015/ 20.02.2022	Active, not recruiting	NCT02393690
Surufatinib/ Toripalimab ^1^	ND	II	ND	01.10.2020/ 30.09.2022	Not yet recruiting	NCT04524884
Dabrafenib	Dabrafenib/ Lenvatinib	II	*BRAF*	07.11.2012/ 31.12.2021	Active, not recruiting	NCT01723202
Trametinib/I^131^	Trametinib/ Dabrafenib/ I^131^	ND	*RAS* (Trametinib/I^131^) or *BRAF* (Trametinib/ Dabrafenib/I^131^)	ND	Ongoing	NCT03244956 (MERAIODE)
PDR001 ^1^/ Trametinib (Cohort A) or Dabrafenib (Cohort B)	ND	II	Cohort A: *BRAF* wt Cohort B: *BRAF-*mutant, resistant to previous *BRAF* inhibitors	02.09.2020/ 02.09.2022	Recruiting	NCT04544111
Vemurafenib/ Copanlisib ^5^	ND	I	*BRAF* V600E	26.06.2020/ 06.2022	Recruiting	NCT04462471

^1^ Anti-PD-1 monoclonal antibody; ^2^ anti-cytotoxic T-lymphocyte antigen-4 (CTLA-4) monoclonal antibody; ^3^ anti-receptor activator of nuclear factor κ-Β ligand (RANKL) monoclonal antibody; ^4^ mTOR inhibitor; ^5^ phosphatidylinositol 3-kinase (PI3K) inhibitor; wt, wild type; ND, not defined.

**Table 3:** Kinase inhibitors currently investigated in clinical trials for ATC treatment (registered under ClinicalTrials.gov and clinicaltrialsregister.eu).

Agent (Drug Name)	Alternative/Control Treatment	Study Phase	Specific Inclusion Criteria	Study Start/ Estimated Completion Date	Status	Trial Number
MEK and BRAF inhibitors (combination therapies)						
Dabrafenib/ Trametinib	ND	II	Mutated *BRAF*	22.01.2021/ 01.2026	Recruiting	NCT04739566 (ANAPLAST-NEO)
Dabrafenib/ Trametinib	ND	I	*BRAF* V600E	04.05.2020/ 30.04.2025	Recruiting	NCT03975231
Cobimetinib/ ABM-1310	ABM-1310	I	*BRAF* V600E	16.06.2020/ 12.2021	Recruiting	NCT04190628
RET inhibitors						
Selpercatinib	ND	II	Mutated *RET*	26.02.2021/ 10.09.2024	Recruiting	NCT04759911
TRK inhibitors						
Larotrectinib	ND	II	*NTRK*	30.09.2015/ 30.09.2025	Recruiting	NCT02576431 (NAVIGATE)
MKIs in combination with other agents						
MKI (ND)/ Anti-PD-1 antibody (ND)	ND	II	Arm C: ATC	30.12.2019/ 30.06.2023	Recruiting	NCT04521348
Cabozantinib/ Atezolizumab ^1^	ND	II	ND	07.10.2020/ 03.2024	Recruiting	NCT04400474 (CABETEN)
Lenvatinib/ Pembrolizumab ^1^	ND	II	ND	02.03.2021/ 31.08.2022	Not yet recruiting	NCT04171622
Pazopanib/ Paclitaxel ^2^/IMRT	Placebo/ Paclitaxel ^2^/IMRT	II	ND	28.10.2010/ lack of data	Active, not recruiting	NCT01236547
SKIs in combination with other agents						
Repotrectinib	ND	I	*ALK, ROS1*, *NTRK* rearrangements	27.02.2017/ 12.2022	Recruiting	NCT03093116 (TRIDENT-1)
Cobimetinib/ Atezolizumab ^1^ (/Vemurafenib if *BRAF* V600E) (Cohort A)	Cobimetinib/ Atezolizumab ^1^ (Cohort B)	II	Mutated *BRAF* (Cohort A) vs. *RAS, NF1, NF2* and MAPK pathway proteins at or above *MEK* mutations (Cohort B)	27.07.2017/ 27.07.2023	Recruiting	NCT03181100
Dabrafenib/ Trametinib/ Cemiplimab ^1^	ND	II	*BRAF* V600E	20.01.2020/ 20.06.2022	Recruiting	NCT04238624
Trametinib/ Dabrafenib/ Pembrolizumab ^1^	ND	II	Mutated *BRAF*	24.06.2021/ 30.06.2024	Recruiting	NCT04675710
Trametinib/ Paclitaxel ^2^	ND	I (pilot study)	ND	03.2017/ 09.2023	Recruiting	NCT03085056

^1^ Anti-PD-1 monoclonal antibody; ^2^ taxan; IMRT, intensity-modulated radiation therapy.

**Table 4:** Kinase inhibitors currently investigated in clinical trials for MTC treatment (registered under ClinicalTrials.gov and clinicaltrialsregister.eu).

Agent (Drug Name)	Alternative/Control Treatment	Study Phase	Specific Inclusion Criteria	Study Start/ Estimated Completion Date	Status	Trial Number
MKIs						
Anlotinib	ND	II	ND	01.01.2019/ 01.06.2022	Active, not recruiting	NCT04309136
Cabozantinib	ND	II	Pediatric MTC	08.05.2017/ 01.07.2022	Active, not recruiting	NCT02867592
Cabozantinib 60 mg	Cabozantinib 140 mg	IV	ND	12.2014/ 12.2022	Active, not recruiting	NCT01896479 (EXAMINER)
Ponatinib	ND	II	ND	26.07.2019/ 06.2022	Recruiting	NCT03838692
Regorafenib	ND	II	ND	01.2016/ 10.2022	Recruiting	NCT02657551
Sorafenib	ND	II	Hereditary vs. sporadic MTC	05.10.2006/ 30.01.2017	Active, not recruiting	NCT00390325
Sunitinib	ND	II	ND	08.08.2006/ 31.12.2016	Active, not recruiting	NCT00381641
Vandetanib	Placebo	III	ND	30.11.2016/ 30.12.2021	Active, not recruiting	NCT00410761
Vandetanib 150 mg	Vandetanib 300 mg	IV	ND	06.2014/ 30.12.2020	Active, not recruiting	NCT01496313
SKIs						
BOS172738	ND	I	*RET*; MTC	12.12.2018/ 06.2022	Active, not recruiting	NCT03780517
Pralsetinib	ND	I/II	*RET*; MTC and PTC	17.03.2017/ 29.02.2024	Recruiting	NCT03037385 (ARROW)
TPX-0046	ND	I/II	*RET*; MTC and other TCs	16.12.2019/ 03.2025	Recruiting	NCT04161391
Selpercatinib	ND	II	*RET*; MTC and other TCs	16.03.2020/ 20.10.2025	Active, not recruiting	NCT04280081 (LIBRETTO-321)
Selpercatinib	ND	I/II	*RET*; MTC and other TCs	02.05.2017/ 21.11.2023	Recruiting	NCT03157128 (LIBRETTO-001)
Selpercatinib	ND	II	*RET*; MTC	26.02.2021/ 10.09.2024	Recruiting	2017-000800-59
Kinase inhibitors in combination with other agents						
MKIs						
MKI (not precised)/ Anti-PD-1 antibody (not precised)	ND	II	Arm B: MTC	30.12.2019/ 30.06.2023	Recruiting	NCT04521348
Apatinib/ Camrelizumab ^1^	ND	II	ND	01.12.2020/ 31.12.2023	Not yet recruiting	NCT04612894
SKIs vs. MKIs						
Pralsetinib	Cabozantinib or Vandetanib	III	Mutated *RET*	01.09.2021/ 15.04.2028	Not yet recruiting	NCT04760288 (AcceleRET-MTC)
Selpercatinib	Cabozantinib or Vandetanib	III	Mutated *RET*	30.12.2019/ 30.06.2023	Recruiting	NCT04211337 (LIBRETTO-531) 2019-001978-28

^1^ Anti-PD-1 monoclonal antibody.

**Table 5:** Most common adverse events (AEs) reported for kinase inhibitors in TC treatment over the past two years.

Research	Study Phase	Agent (Dose or Group)	No of Patients: Total/ Dropouts due to AEs	Dose Reduction (No of Patients)	ORR	DCR	CR	PR	SD	PD	OS (Months)	PFS (Months)	All-Grade Most Common AEs (%)
MKIs													
Li et al. [161]	IIB	Anlotinib vs. placebo vs. open label	62 vs. 29 vs. 12	10 vs. 1 vs. 1	48% vs. 0% vs. 33%	89% vs. 86% vs. 83%	ND	ND	ND	ND	50 vs. 19 vs. ND	21 vs. 11 vs. 15	PPES (63% vs. 10% vs. ND) Proteinuria (61% vs. 10% vs. ND) Hypertriglyceridemia (48% vs. 24% vs. ND)
Lin et al. [119]	II	Apatinib (500 mg vs. 750 mg)	10 vs. 10	5 vs. 9	70% vs. 90%	90% vs. 100%	0% vs. 0%	70% vs. 90%	20% vs. 10%	ND	34 vs. 52	14 vs. 35	PPES (95%) Proteinuria (90%) HT (80%)
Brose et al. [122]	III	Cabozantinib vs. placebo	125/6 vs. 62/0	70 vs. 3	35% vs. 2%	43% vs. 16%	0% vs. 0%	9% vs. 0%	61% vs. 34%	6% vs. 50%	ND	6 vs. 2	Diarrhoea (51% vs. 3%) PPES (45% vs. 0%) HT (28% vs. 5%)
Lin et al. [121]	II	Donafenib (200 mg vs. 300 mg)	17/1 vs. 18/3	8 vs. 13	13% vs. 13%	100% vs. 100%	ND	13% vs. 13%	88% vs. 87%	ND	ND	9 vs. 15	PPES (88% vs. 78%) Alopecia (65% vs. 78%) HT (47% vs. 44%)
Wirth et al. [146]	II	Lenvatinib	34/6	14	3%	53%	0%	3%	50%	27%	3	3	HT (56%) Decreased appetite (29%) Fatigue (29%) Stomatitis (29%)
Takahashi et al. [124]	ND	Lenvatinib (DTC or ATC or MTC)	442 or 124 or 28	105 or 37 or 6	59% or 44% or 45%	92% or 76% or 100%	3% or 3% or 5%	57% or 41% or 40%	33% vs. 32% vs. 55%	4% vs. 24% vs. 0%	ND or ND or 4	ND	HT (79% or 70% or 64%) Proteinuria (43% or 30% or 39%) PPES (39% or 26% or 50%)
Song et al. [126]	ND	Lenvatinib	43/6	30	ND	98%	ND	65%	56%	2%	ND	22	Fatigue or asthenia (72%) Diarrhoea (67%) HT (63%) Proteinuria (58%)
Porcelli et al. [172]	ND	Lenvatinib	23/3	23	ND	ND	ND	26%	61%	4%	46	25	HT (78%) Fatigue (74%) Weight loss (65%)
Matrone et al. [160]	ND	Lenvatinib	9	7	36%	80%	ND	11%	89%	ND	ND	ND	Asthenia (50%) Nausea (40%) Diarrhoea (30%) Weight loss (30%)
Ito et al. [127]	ND	Sorafenib vs. Lenvatinib	21 vs. 18	ND	ND	ND	ND	22% vs. 65%	72% vs. 24%	6% vs. 2%	ND	ND	PPES (81% vs. 39%) HT (19% vs. 94%) Thrombocytopenia (ND vs. 50%) Hypocalcemia (38% vs. ND) Proteinuria (33% vs 33%)
Koehler et al. [125]	ND	Sorafenib vs. Lenvatinib vs. Pazopanib	33/14 vs. 53/23 vs. 15/4	16 vs. 31 vs. 6	18% vs. 70% vs. 60%	ND	0% vs. 2% vs. 7%	18% vs. 68% vs. 53%	21% vs. 9% vs. 13%	ND	37 vs. 47 vs. 34	9 vs. 12 vs. 12	Loss of appetite/weight (46% vs. 49% vs. 25%) Diarrhoea (43% vs. 44% vs. 42%) Fatigue (ND vs. 44% vs. 29%)
Valerio et al. [158]	ND	Vandetanib (short-term vs. long-term treatment)	25 vs. 54	ND	ND	ND	ND	ND vs. 26%	ND vs. 72%	ND vs. 2%	ND	47 vs. 87	Hypothyroidism (92% vs. 100%) Rash (28% vs. 48%) Diarrhoea (12% vs. 39%)
SKIs													
Salama et al. [134]	II	Dabrafenib and Trametinib	29	ND	38%	76	ND	38%	38%	7%	29	11	Fatigue (74%) Nausea (57%) Chills (54%)
Doebele et al. [139]	I /II	Entrectinib	68/3	27	57%	ND	7%	50%	17%	7%	21	11	Dysgeusia (47%) Fatigue (35%) Constipation (28%)
Desai et al. [138]	I	Lifirafenib (dose escalation vs. dose expansion)	35/5 vs. 96/19	ND	ND	ND	ND	ND	ND	ND	ND	ND	Fatigue (69% vs. 49%) Acneiform dermatitis (43% vs. 18%) Decreased appetite (40% vs. 36%) Constipation (40% vs. 27%)
Subbiah et al. [162]	I/II	Pralsetinib (*RET*m MTC previously treated or *RET*m MTC with no previous treatment or *RET*f TC)	55 or 21 or 9	5 (and 8 deaths)	60% or 71% or 89%	93% or 100% or 100%	2% or 5% or 0%	58% or 67% or 89%	33% vs. 29% vs. 11%	4% vs. 0% vs. 0%	17 or 19 or 16	15 or 15 or 13	Neutropenia (34%) Decreased WBC count (34%) Increased AST level (34%)
Wirth et al. [163]	I/II	Selpercatinib (*RET*m MTC previously treated or *RET*m MTC with no previous treatment or *RET*f TC previously treated)	55 or 88 or 19	ND	62% or 71% or 58%	69% or 67% or 58%	5% or 3% or 0%	56% or 67% or 58%	29% vs. 27% vs. 37%	5% vs. 0% vs. 0%	ND	27 or 24 or ND	Dry mouth (46%) HT (43%) Diarrhoea (38%) Fatigue (38%)

AST, aspartate aminotransferase; CR, complete response; DCR, disease control rate; HT, hypertension; ORR, overall response rate; OS, overall survival; PD, progressive disease; PFS, progression-free survival; PPES, palmar-plantar erythrodysesthesia syndrome; PR, partial response; *RET*f, *RET* fusion-positive; *RET*m, *RET* mutation-positive; SD, stable disease; WBC, white blood cell.

## Abbreviations

AEs	adverse events
ALK	anaplastic lymphoma kinase
AST	aspartate aminotransferase
ATA	American Thyroid Association
ATC	anaplastic thyroid cancer
BMI	body mass index
BRAF	B-Raf proto-oncogene
CT	computer tomography
CTLA-4	cytotoxic T-lymphocyte antigen-4
DeTC	dedifferentiated thyroid cancer
DIT	diiodotyrosine
DTC	differentiated thyroid cancer
EBRT	external beam radiation therapy
eIF1A	eukaryotic translation initiation factor 1A
EMA	European Medical Agency
FAP	familial adenomatous polyposis
FDA	Food and Drug Administration
^18^FDG	^18^F-fluoro-2-deoxy-D-glucose
FNAB	fine-needle aspiration biopsy
FTC	follicular thyroid carcinoma
FVPTC	follicular variant of PTC
GTP	guanosine triphosphate
HCC	Hürtle cell carcinoma
HNHA	N-hydroxy-7-(2-naphthylthio) heptanamide
HT	hypertension
IDH1	isocitrate dehydrogenase 1
IDH2	isocitrate dehydrogenase 2
IMRT	intensity-modulated radiation therapy
KIs	kinase inhibitors
MAPK	mitogen-activated protein kinase
MEN	multiple endocrine neoplasia
MEN2	multiple endocrine neoplasia type 2
MIT	monoiodotyrosine
MKIs	multi-kinase inhibitors
MRI	magnetic resonance imaging
MTC	medullary thyroid cancer
ND	not defined
NIS	sodium iodide symporter
ORR	overall response rate
OS	overall survival
PAX8	paired box 8
PD	progressive disease
PDCT	poorly differentiated thyroid carcinoma
PET	positron emission tomography
PFS	progression-free survival
PI3K	phosphatidylinositol 3-kinase
PIK3CA	phosphatidylinositol-4,5-bisphosphate 3-kinase
PLC-γ	phospholipase C
PPARγ	peroxisome proliferator activated receptor γ
PPES	palmar-plantar erythrodysesthesia syndrome
PR	partial response
PTC	papillary thyroid carcinoma
RAI	radioactive iodine; radioiodine
RAIR	radioactive iodine refractory; radioiodine refractory
RANKL	receptor activator of nuclear factor κ-Β ligand
*RET*f	*RET* fusion-positive
*RET*m	*RET* mutation-positive
RTK	receptor tyrosine kinase
RWS	Real-Word Studies
SD	stable disease
SKIs	specific kinase inhibitors
T3	triiodothyronine
T4	thyroxine
TBSRTC	The Bethesda System for Reporting Thyroid Cytopathology
TC	thyroid cancer
TCGA	The Cancer Genome Atlas
TERT	telomerase reverse transcriptase
Tg	thyroglobulin
THs	thyroid hormones
TPO	thyroid peroxidase
TRK	tropomyosin receptor kinase
TSH	thyroid-stimulating hormone
USG	ultrasonography
WBC	white blood cell
WHO	World Health Organization
wt	wild type
